# Immune landscape and oncobiota in HPV-Associated Colorectal Cancer: an explorative study

**DOI:** 10.1007/s10238-023-01165-3

**Published:** 2023-08-23

**Authors:** Maria Raffaella Ambrosio, Elena Niccolai, Federica Petrelli, Leandro Di Gloria, Gloria Bertacca, Andrea Giusti, Simone Baldi, Andrea Cavazzana, Matteo Palmeri, Bruno Perotti, Matteo Ramazzotti, Marco Arganini, Amedeo Amedei

**Affiliations:** 1 Pathology Unit, Azienda USL Toscana Nord Ovest, Pisa, Italy; 2 Department of Clinical and Experimental Medicine, University of Florence, Largo Brambilla 3, 50134 Florence, Italy; 3 Department of Biomedical, Experimental and Clinical Sciences, “Mario Serio” University of Florence, Florence, Italy; 4 Clinical Chemical Analysis and Immuno Allergology Department, Azienda USL Toscana Nord Ovest, Pisa, Italy; 5 Surgery Unit, Ospedale Unico Versilia, Azienda USL Toscana Nord Ovest, Pisa, Italy; 6 Internal Interdisciplinary Medicine Unit, Careggi University Hospital, 50134 Florence, Italy

**Keywords:** Human Papillomavirus, Colorectal cancer, Microbiota, Oncobiota, Immunity, Immune evasion, *Bacteroides*

## Abstract

Worldwide more than 550,000 new patients suffering from malignant tumors are associated with human papillomaviruses (HPV) infection. However, only a small portion of patients infected progress to cancer, suggesting that other factors other than HPV may play a role. Some studies have investigated HPV infection in colorectal cancer (CRC) with discordant results; moreover, the role of HPV in CRC development is still unknown. We investigated HPV infection in 50 CRC from different regions, excluding the anal one, by immunohistochemistry (IHC), real-time PCR and RNA-seq. For each patient, we studied the tumor microenvironment in neoplastic and matched non-neoplastic samples, and we compared the tumor-infiltrating immune cell phenotypes among HPV-positive and negative samples. Finally, we compared the CRC-associated microbiota in HPV-positive and negative neoplastic samples by 16S rRNA sequencing. HPV infection was identified in 20% of CRC from the right side (caecum, ascending and transverse colon) and in 40% from the left side (descending colon and rectum). In all HPV-positive CRCs we found no expression of p53 and RB, thus suggesting HPV involvement in tumorigenesis. As far as the tumor microenvironment is concerned, in HPV-related cancers we observed a neoplastic environment with a reduced immune surveillance but an enhanced cytotoxic response by lymphocytes. HPV-positive and -negative CRC showed a different microbiota with lack of species normally found in CRC in the HPV-positive ones. Our results support the carcinogenic significance of HPV in CRC, suggesting a role of HPV in modulating the tumor immune microenvironment.

**Supplementary Information:**

The online version contains supplementary material available at 10.1007/s10238-023-01165-3.

## Introduction

Human papillomavirus (HPV) infection with high-risk subtypes (i.e. HPV 16, 18, 31, 33, 35, 45, 52, 58) is recognized as one of the major causes of infection-related cancers worldwide [1]. In detail, the International Agency for Research on Cancer has established strong evidence for the causal HPV etiology in cancer of the cervix uteri (95–99%), anus (88–94%), vagina (65–90%), vulva and penis (40–50%), and oropharynx (30%) [2]. Of note, although being of different histotypes (squamous cell carcinoma and adenocarcinoma) and arising in different organs, all these tumors share many common and immunological properties [3].

Usually, the HPV infections are cleared by the host immune system within 1–2 years but, for unknown reasons, in 10–15% of patients, infection does not resolve and persists, eventually leading to invasive cancer if not timely and properly managed and treated [4]. Therefore, HPV presence alone is insufficient to account for neoplastic development. It is conceivable that other factors, specific to each individual, likely play a role in HPV integration and so, progression to cancer [5].

In this regard, a local and efficient immune response at the right time seems to be crucial to eliminate the invading pathogen[6]. On the contrary, the infection persists and may hesitate in cancer development [7]. This occurrence takes place also in non-immunosuppressed patients and can require many years [8]. Over this period, the virus itself employs a diverse array of mechanisms that could establish a state of immune dysregulation [9]. The impairment of immune response in infected cells affects their capacity to alert immune cells, resulting in an overall immune suppressive environment cancer–promoting [10]. Another important aspect of HPV-related cancers, concerning the access routes of the virus, is the potential shifting in the commensal bacteria and the consequent effects on the innate patients’ immunity [11]. Dysbiosis may affect immune response allowing HPV to become more aggressive in its capacity to infect more cells and integrate [12].

However, while the biological mechanisms underlying the malignant transformation of HPV-infected cells are well established (*i.e.* E6 and E7-mediated down-regulation of p53 and Rb), our knowledge of the reasons why only a small percentage of people develop cancer is still limited [6, 13]. Due to the great prevalence of the virus in the global population and the rising rates of colorectal cancer (CRC) in those under 50, the scientific community has recently begun to pay more attention to a possible role for HPV in CRC. This subset of population includes sexually active individuals showing an even higher burden of HPV infection. However, the link is still theoretical and a consensus on this association has not yet been found. In fact, the more recent studies reported a prevalence of HPV in CRCs ranging from 31 to 53% [14–16].

In this scenario, we planned a study to establish a pathogenetic link between HPV and CRC excluding the anal site for which the pathogenetic link has been already proved. To shed new lights on the mechanisms favoring the evolution towards an invasive tumor, we focused on the immune landscape of HPV-related CRC and on the resident microbiota composition, finding a peculiar immunologic pattern and a distinct oncobiota in HPV positive and negative CRC samples.

## Methods

### Patients

This is a retrospective study enrolling a total of 50 patients consecutively treated by surgery for CRC of caecum, ascending, transverse and descending colon, rectum. The patients had been diagnosed by expert gastrointestinal pathologists according to the World Health Organization (WHO) classification [17]. The study was conducted in accordance with the Declaration of Helsinki and approved by the Ethic Committee of the Azienda Toscana Nord Ovest. The needing of informed written consent was waved due to the retrospective nature of the study.

### Immunohistochemistry and scoring

Immunohistochemical (IHC) analyses were performed on all the neoplastic samples and 50 matched non-neoplastic controls from the same patients, using standard procedures, on automated staining system, as previously described [1]. The sections were evaluated by two pathologists in a blinded fashion. Specifically, we examined different immune cells, namely cytotoxic T-lymphocytes (CTL-expressing CD8), helper T-cells (TH-expressing CD4), antigen presenting cells (APC-expressing CD21) and natural killer cells (NK-expressing CD56). To detect the presence of T-exhausted (T-exh) phenotype, we defined the percentage of CD8-positive lymphocytes co-expressing PD-1; for the T-regulatory phenotype the percentage of CD4-positive cells co-expressing CD25; for the M2-phenotype the ratio between CD163 and CD68 positive tumor associated macrophages (TAM). Each slide was screened at 20× magnification, then we selected the hot-spot areas and we evaluated the mean of the expression of all the antibodies in three different high power (40x) fields. Already known scoring system, based on previous papers were applied [1, 18].

### HPV identification and genotyping

A commercial kit (HPV sign; Diatech, Jesi, Italy) was used to detect the presence of virus by real-time PCR of L1 region of HPV and the human beta-globin gene as the internal control as previously described [19]. The reverse specific primers for HPV detection were biotinilated. The thermal profile performed on a RotorGeneTM 6000 instrument (Corbett, New South Wales, Australia) was 95 °C for 3 min, 50 cycles at 95 °C for 0.5 min; 44 °C for 0.5 min; 72 °C for 0.5 min; followed by a final melting from 72 °C to 95 °C with 1 °C increment at each step. The obtained melting curve was analysed in order to assign the positivity of the sample.

The byotinilated HPV positive PCR products were subjected to pyrosequencing using a commercial kit (HPV sign; Diatech, Jesi, Italy) according to the manufacturer’s instructions, as previously described [19]. After some steps of purification from the reaction mixture with the PyroMark Q96 ID system (Qiagen, Hilden, Germany) according to themanufacturer’s instructions, the DNA was denaturated by heating and added to sequence HPV primers. The sequencing reaction was performed in the presence of substrate and enzyme by dispensation of dATPαS, dCTP, dGTP and dTTP for 15 cycles on PyroMark Q96 ID system (Qiagen, Hilden, Germany). The specific HPV sequences were 30 bp length and were compared with those contained in the library of IdentiFireTM software that assigned the genotype by a score of percentage of identity. [1].

### Microbiota characterization

We analyzed the CRC-associated microbiota in 10 HPV-positive and 10-negative neoplastic samples. The first few scrolls of the FFPE blocks for each sample were discarded, to reduce environmental contamination. Eight or ten sections, each having a thickness of up to 10 m and a surface area of up to 250 mm^2^, were then obtained by a microtome and collected into sterile 2 ml centrifuge tubes. Between patient samples, the microtome was cleaned, and sterile swabs were periodically used to check the machinery. Genomic DNA was extracted using the QIAmp DNA FFPE Advanced Kit (Qiagen, Hilden, Germany) which allows the purification of genomic DNA from tissue samples fixed in formalin and embedded in paraffin, for efficient high-throughput sequencing. According to the Illumina 16S Metagenomic Sequencing Library Preparation protocol, amplicons of the variable V3–V4 region of the bacterial 16 s rRNA gene were sequenced in paired-end (2 × 300 cycles) on the Illumina MiSeq platform by the IGA Technology Services (Udine, Italy). Demultiplexed sequence reads were processed using QIIME2 2021.4. The sequencing primers and the reads without primers were removed using Cutadapt tool. DADA2 was used to perform pairedend reads filtering, merging and chimeras removal steps after trimming low quality nucleotides from both forward and reverse reads. Hence, ASVs (amplicon sequence variants) were generated and the taxonomic assignments have been performed through a scikit-learn multinomial naïve Bayes classifier trained on SILVA database (release 138) V3-V4 iper-variable region. PICRUST2 was utilized to predict the expressed METACYC pathways through SEPP placement algorithm.

### Mutation screening of targeted cancer-associated genes

Next generation sequencing (NGS) analysis, using the commercial kit “Miriapod NGS-IL” (Diatech) on MiSeq Illumina Platform, was carried out on HPV-positive tumor samples to identify possible *P53* and *RB* gene mutations.

### Statistical analysis

Statistical analysis of the IHC results was carried out by using commercially available statistical soft-ware (SPSS 24.0 for Windows SPSS Inc., Chicago, IL, USA) to calculate the association of epidemiologic and clinico-pathological characteristics between the two groups. Chi-squared and Fisher’s exact test were performed for descriptive statistics. The Mann–Whitney-U test was used to compare continuous variables not normally distributed. All *p* values were two-sided with *p* < 0.05 considered statistically significant.

Statistical analyses on the bacterial communities were performed in R 4.1 with the help of the packages phyloseq 1.36.0, DESeq2 1.32.0 and other packages satisfying their dependencies, as vegan 2.5-7. Packages ggplot2 3.3.5 and dendextend 1.15.1 were used to plot data and results.

Shannon and Pielou’s evenness indices and Observed ASV richness, were used to estimate bacterial diversity in each sample using the function estimate_richness from phyloseq. The evenness index was calculated using the formula E = S/log(R), where S is the Shannon diversity index and R is the Observed ASV richness in the sample; differences in all indices were tested using the Mann–Whitney test. PCoAs were performed on proportional count data of each sample adjusted with square root transformation while the differential analysis of abundances was performed with DESeq2 on raw count data at the different taxonomic ranks. Differential abundances of predicted pathways were determined and displayed using LefSe (Linear discriminant analysis effect Size) tool on Galaxy platform.

Spearman correlation coefficients was computed to assess association between variables. p-values were corrected for multiple comparisons using the Benjamini–Hochberg FDR procedure.

## Results

### HPV detection and oncogenicity

**Fig. 1:**
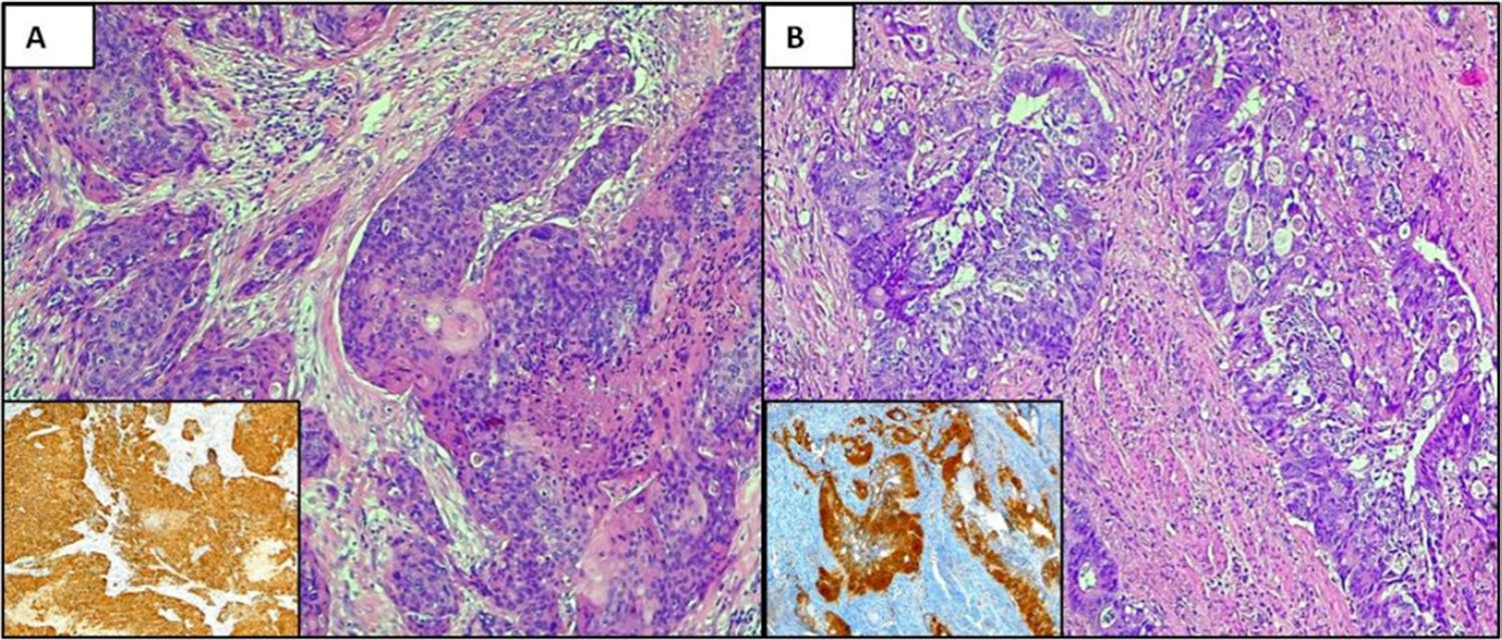
Expression of p16 in CRC. An example of squamous cell carcinoma (**A**) and adenocarcinoma **B** of the colon showing strong and diffuse expression of p16 in neoplastic cells (A inset, B inset). A, B Haematoxylin and eosin stain; A inset, B inset, p16 stain. Original magnification (O.M.): A, A inset, B, B inset O.M. 10x

HPV was searched in neoplastic and non-neoplastic tissue of each patient by IHC for p16 and PCR assay. The clinical data and the pathological features of our cohort of patients are reported in Table 1. IHC for p16 has been previously reported as a useful surrogate biomarker of HPV integration in cervical and head and neck cancer [20, 21]. By immunohistochemistry, strong expression was detected in 10 out of 25 (40%) CRC of the right side and in 18 (70%) from the left one (Fig. 1A, B). Specifically, p16 positivity was identified in neoplastic cells, whereas non-neoplastic tissues did not show p16 expression.

**Table 1:** Patients’ clinical and pathological data

N	GENDER	AGE	SITE	HISTOTYPE	GRADING	STAGE	MSI
1 PV	F	89	Rectum	Squamous carcinoma	G2	pT4N0	pMMR
2 PMG	F	53	Descending colon	Squamous carcinoma	G2	pT3N0	pMMR
3 BA	F	57	Rectum	Squamous carcinoma	G3	pT3N0	pMMR
4 LA	F	44	Rectum	Adenocarcinoma, NOS	G2	pT3N0	pMMR
5 SA	F	50	Ascending colon	Adenocarcinoma, NOS	G2	pT4N0	pMMR
6 GG	F	80	Rectum	Basaloid carcinoma	G1	pT2N0	pMMR
7 MA	M	49	Ascending colon	Adenocarcinoma, NOS	G2	pT2N0	pMMR
8 SF	M	63	Descending colon	Adenocarcinoma, NOS	G3	pT3N2a	pMMR
9 CF	M	74	Ascending colon	Adenocarcinoma, NOS	G3	pT4aN2a	pMMR
10 FM	F	83	Descending colon	Adenocarcinoma, NOS	G3	pT3N2b	pMMR
11	F	55	Rectum	Squamous carcinoma	G2	pT3N0	pMMR
12	M	58	Rectum	Squamous carcinoma	G2	pT3N0	pMMR
13	F	81	Rectum	Squamous carcinoma	G3	pT4N0	pMMR
14	M	74	Ascending colon	Adenocarcinoma, NOS	G2	pT3N0	pMMR
15	M	67	Transverse colon	Adenocarcinoma, NOS	G2	pT4N0	pMMR
16 LA	M	65	Ascending colon	Adenocarcinoma, NOS	G3	pT3N2a	dMRR (*BRAF* V600E mutation)
17 BL	F	59	Descending colon	Adenocarcinoma, NOS	G3	pT4N2a	pMMR
18 RL	M	51	Descending colon	Adenocarcinoma, NOS	G2	pT3N0	pMMR
19 BS	M	54	Rectum	Adenocarcinoma, NOS	G2	pT2N0	pMMR
20 IA	F	58	Descending colon	Adenocarcinoma, NOS	G2	pT3N0	pMMR
21 CV	F	58	Descending colon	Adenocarcinoma, NOS	G2	pT1N0	pMMR
22 FFCC	F	47	Descending colon	Adenocarcinoma, NOS	G2	pT3N0	pMMR
23 BC	F	48	Rectum	Adenocarcinoma, NOS	G2	pT4N2b	pMMR
24 MTF	M	82	Ascending colon	Adenocarcinoma, NOS	G3	pT4aN0	dMMR (*BRAF* V600E mutation)
25 BA	F	81	Ascending colon	Adenocarcinoma, NOS	G2	pT4aN2b	pMMR
26	F	66	Descending colon	Adenocarcinoma, NOS	G2	pT4N0	dMMR (*BRAF* V600E mutation)
27	F	78	Ascending colon	Medullary carcinoma	n.a	pT3N0	pMMR
28	F	69	Ascending colon	Mucinous adenocarcinoma	G2	pT4N2a	dMMR (*BRAF* V600E mutation)
29	M	81	Descending colon	Adenocarcinoma, NOS	G2	pT3N0	pMMR
30	M	65	Ascending colon	Adenoma-like adenocarcinoma, NOS	G1	pT2N0	pMMR
31	F	82	Transverse colon	Adenocarcinoma, NOS	G3	pT3N2a	dMMR (*BRAF* V600E mutation)
32	M	85	Descending colon	Adenocarcinoma, NOS	G3	pT4aN2a	pMMR
33	M	91	Descending colon	Micropapillary adenocarcinoma, NOS	n.a	pT3N2b	pMMR
34	M	72	Ascending colon	Mucinous adenocarcinoma, NOS	G3	pT4aN2b	dMMR (*BRAF* V600E mutation)
35	F	77	Ascending colon	Adenocarcinoma, NOS	G2	pT3N0	pMMR
36	M	65	Rectum	Squamous cell carcinoma	G2	pT4N0	pMMR
37	F	64	Descending colon	Adenocarcinoma, NOS	G3	pT3N0	pMMR
38	M	69	Ascending colon	Adenocarcinoma, NOS	G2	pT2N0	pMMR
39	F	76	Ascending colon	Adenocarcinoma, NOS	G2	pT3N2a	dMMR (*BRAF* V600E mutation)
40	F	65	Rectum	Squamous cell carcinoma	G2	pT3N0	pMMR
41	F	61	Ascending colon	Adenocarcinoma, NOS	G2	pT3N2b	pMMR
42	M	58	Ascending colon	Signet ring cell carcinoma, NOS	n.a	pT4aN2c	dMMR (*BRAF* V600E mutation)
43	M	73	Descending colon	Adenocarcinoma, NOS	G3	pT3N0	pMMR
44	M	75	Ascending colon	Adenocarcinoma, NOS	G2	pT4N0	pMMR
45	M	80	Caecum	Adenocarcinoma, NOS	G2	pT3N0	pMMR
46	M	79	Transverse colon	Adenocarcinoma, NOS	G2	pT4aN1a	pMMR
47	F	76	Ascending colon	Adenocarcinoma, NOS	G2	pT3N2a	pMMR
48	F	74	Caecum	Mucinous carcinoma, NOS	G2	pT4aN1a	dMMR (*BRAF* V600E mutation)
49	F	73	Ascending colon	Adenocarcinoma, NOS	G3	pT4aN2a	dMMR (*BRAF* V600E mutation)
50	M	83	Transverse colon	Adenocarcinoma, NOS	G3	pT3N2b	pMMR

By PCR assay, HPV-DNA was found in 5 out of 25 (20%) CRC from caecum, ascending and transverse colon, and in 10 out of 25 (40%) from descending colon and rectum. No differences in terms of age, gender, grading, staging were identified. Most of squamous cell carcinomas (*n* = *6*) were HPV-related, whereas no special histotypes demonstrated the viral DNA. Interestingly, all HPV-positive tumors were proficient for the mismatch repair proteins (pMMR) with a lower rate of mutations by NGS.

As HPV strains was not identified by PCR in all p16-positive cases, one could affirm that in gastrointestinal tract p16 protein expression should not be used as a surrogate for HPV infection.

Sequence analysis of PCR products sustained the specificity of the PCR results and identified HPV 16 (*n* = 9) and 18 (*n* = 6) genotypes as the only strain present in HPV-DNA positive samples.

The expression of p53 and Rb was absent in the neoplastic tissues of HPV positive cases. Moreover, by NGS analysis, no *P53* and/or *RB* inactivating gene mutations accounting for the lack of protein expression, was detected. These findings support the view that HPV itself might have induced the down-regulation of p53 and Rbin the infected patients.

### Cancer-associated microbiota in HPV-positive and –negative patients

**Fig. 2:**
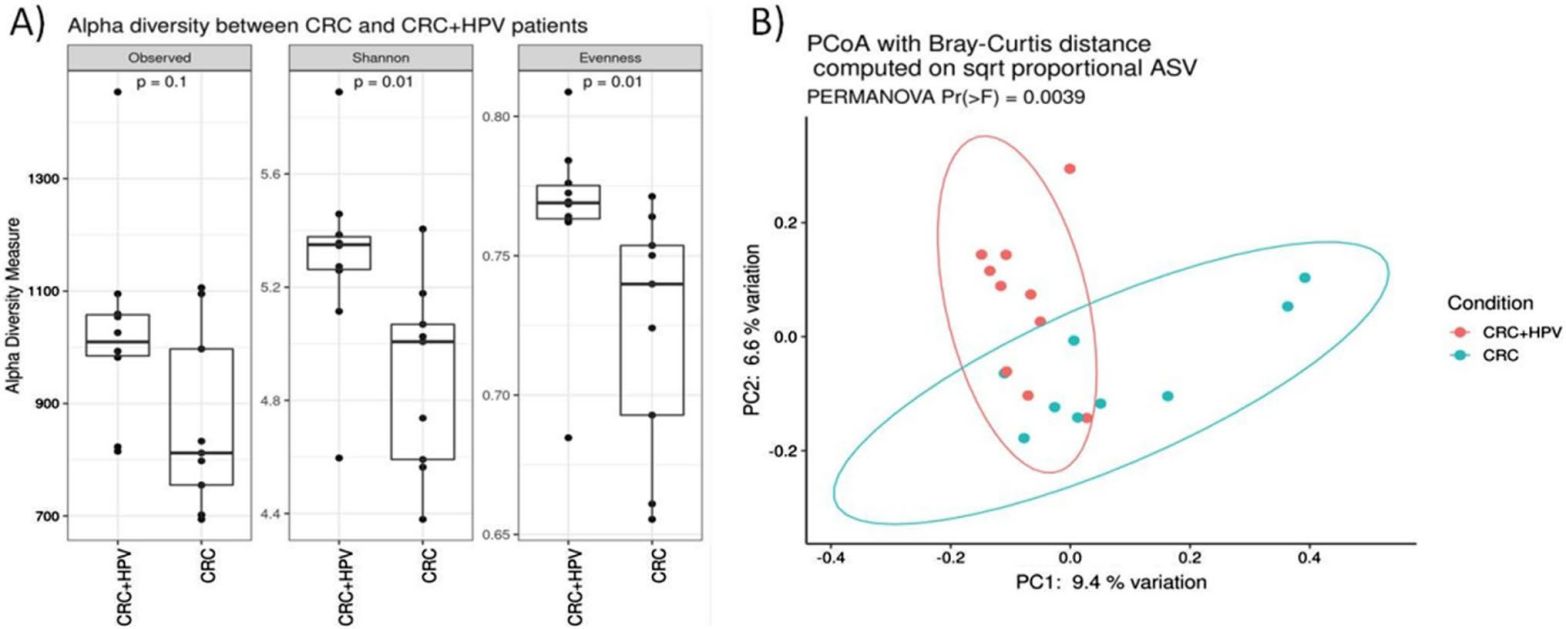
Alpha and Beta diversity analysis between HPV-positive and HPV-negative CRC samples. **A** Boxplots showcasing alpha diversity indices (Observed, Shannon index, Evenness). Statistical differences were evaluated using paired Wilcoxon signed-rank test. P-values less than 0.05 were considered statistically significant. **B** Principal coordinates analysis (PCoA) according to the Bray–Curtis beta-diversity metric. Results of the permutational multivariate analysis of variance (PERMANOVA) are also shown based on the first two coordinates. Both 2D and 3D representation are provided. **C** Agglomerative cluster analysis using Euclidean distance as metric

The CRC-associated microbiota, in term of community heterogeneity and configuration was different in HPV-positive and -negative samples. In detail, the study of alpha diversity, performed trough the measure of the observed species index (*p* = 0.1), Shannon index (*p* = 0.01), and Evenness (*p* = 0.01), indicated that HPV-positive samples had a higher number of microbial species and a greater diversity respect to the HPV-negative tumors (Fig. 2A). Furthermore, the beta diversity, calculated through PCoA using Bray Curtis distance on sqrt proportional ASV, showed that HPV-positive samples had a different community structure compared to HPV negative (PERMANOVA, Pr(> *F*) = 0.0027, Fig. 2B). Notably, no difference was observed in the overall microbiota structure among samples grouped according to gender, age, cancer type, side (left vs right), and disease stage (data not shown).

**Fig. 3:**
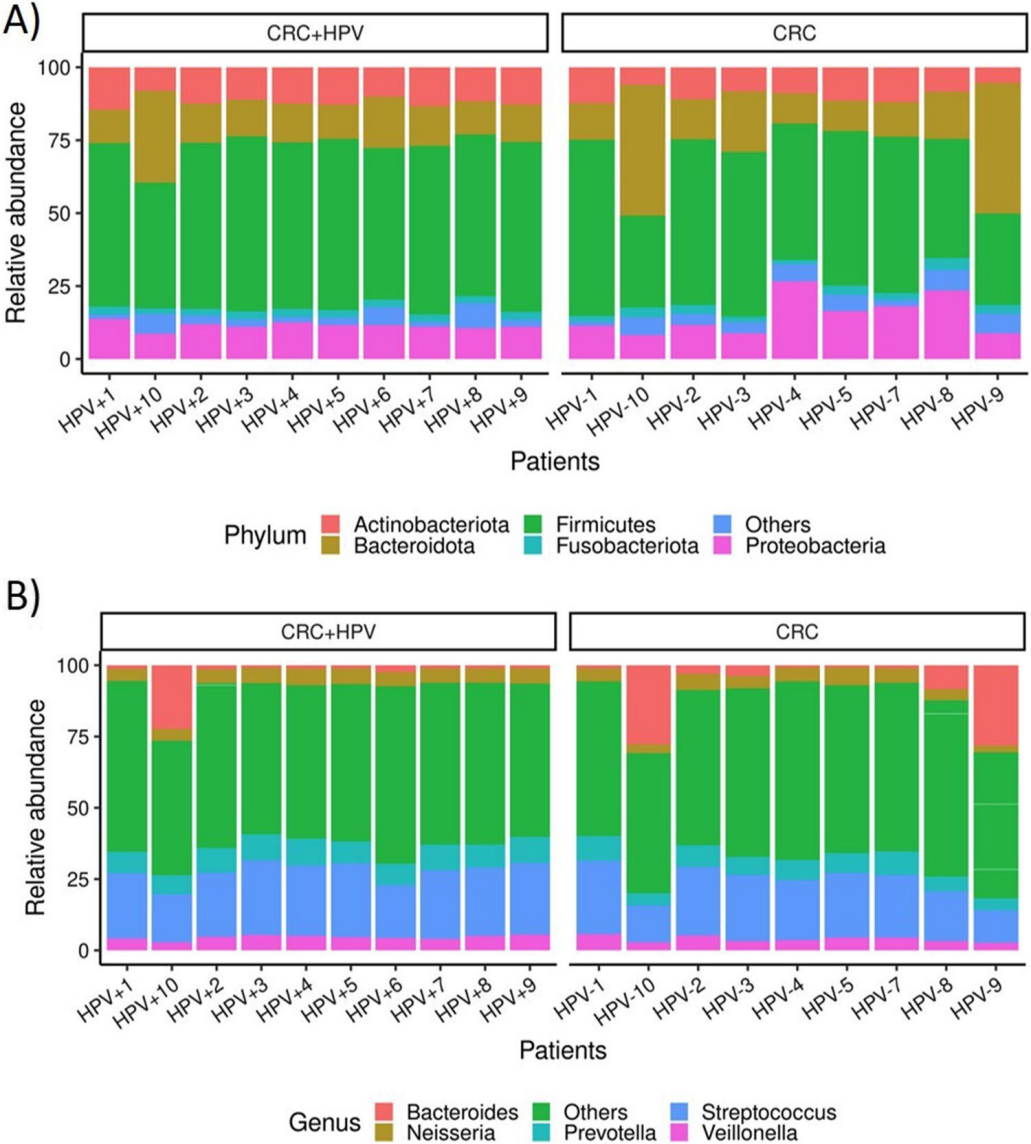
Top five most abundant taxa in HPV-positive and HPV-negative CRC samples. A) Stacked bar plots displaying the average relative abundance of bacterial amplicon sequence variants (ASVs) identified at the phylum (**A**) and genera (**B**) taxonomic level

Regarding the taxonomic composition, the CRC- associated microbiota was dominated by five phyla: Firmicutes, Bacteroidota, Proteobacteria, Actinobacteriota and Fusobacteriota in descending order, and the genera *Streptococcus*, *Prevotella*, *Bacteroides*, *Neisseria*, and *Veillonella*, in all samples (Fig. 3).

**Fig. 4:**
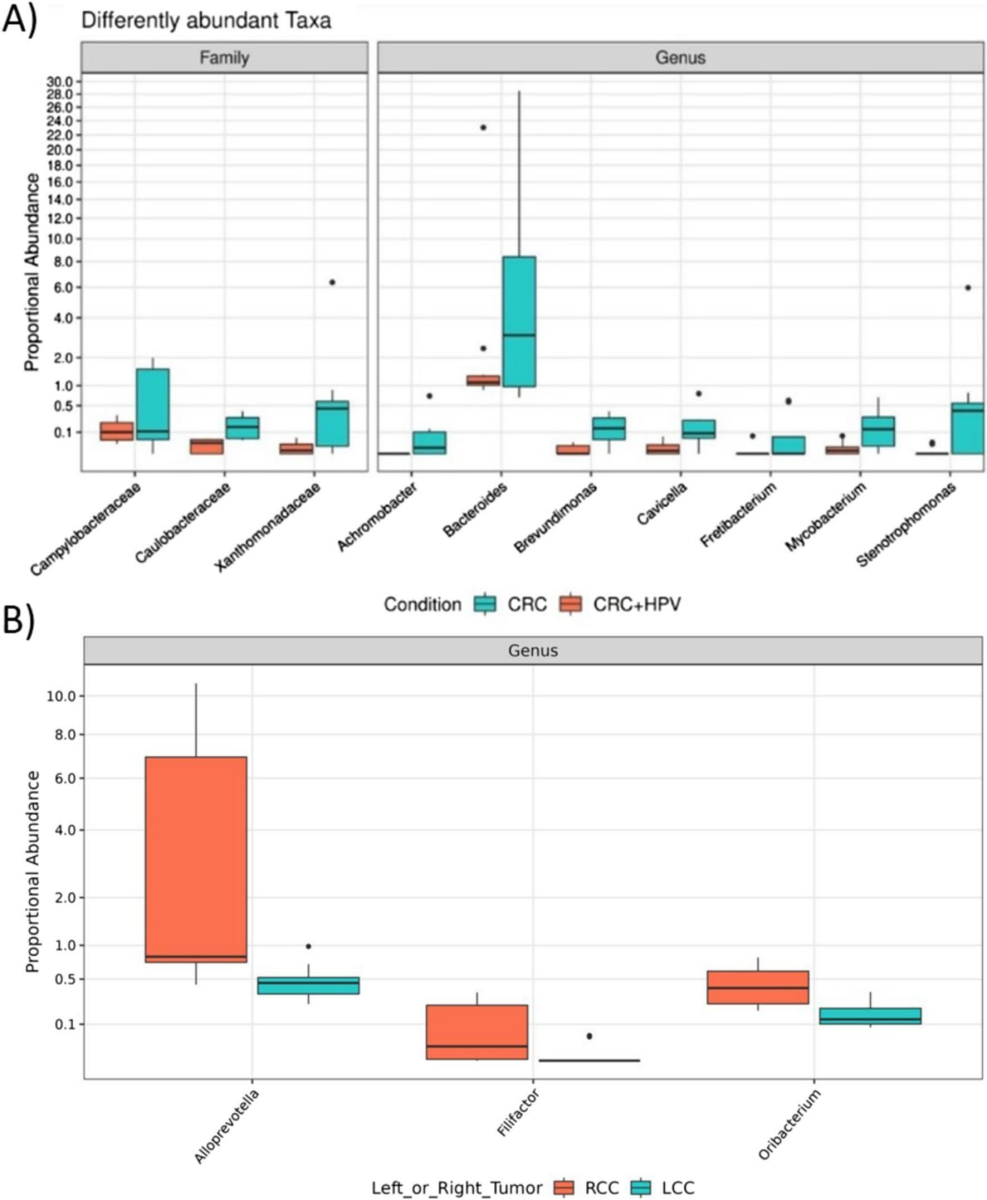
DESeq2 results of the differential abundance taxa in **A** HPV-positive and HPV-negative CRC samples, **B** left (LCC) and right-sides (RCC) colon cancers

Anyway, multivariate analysis has shown that 3 families (Caulobacteraceae, Campylobacteraceae, Xanthomonadaceae) and 7 genera (*Achromobacter*, *Bacteroides*, *Brevundimonas*, *Cavicella*, *Fretibacterium*, *Mycobacterium*, *Stenotrophomonas*) were more abundant in HPV-negative compared to HPV-positive CRC samples (Fig. 4; Table S1).

Of note, we also found a slight difference in the abundance of three genera (*Alloprevotella*, *Filifactor* and *Oribacterium*) in right-sided and left-sided colon cancer samples (Fig. 4B).

To predict the relative abundances of functional categories and to deduce functional capacities microbial differences between the two groups we performed the PICRUST analysis. A total of 42 MetaCyc pathways, especially related to glutamine, glutamate, amino acids and nucleotides biosynthesis, were enriched in HPV-positive CRC-associated microbiota; rather, 10 predicted metabolic pathways were more abundant in HPV-negative CRC- associated microbiota (logLDA score > 2) (Supplementary Fig. 1).

### CRC microenvironment characterization

To highlight potential differences between HPV-positive and -negative samples, we studied the neoplastic and non-neoplastic microenvironment of our patients.

In HPV-associated tumors we identified a dichotomic distribution of lymphoid populations; in detail:

 − in neoplastic cells, higher PD-L1 expression was observed; the neoplastic microenvironment showed PD-1 positivity of the reactive cells, and higher number of Texh and Treg cells. In addition, M2 polarization of TAM was present as defined by the high CD163/CD68 ratio (Supplementary Fig. 2A–D).
 − in matched non-neoplastic samples, higher rate of CTL and TH cells was detected with lower number of Texh and Treg lymphocytes, and M1-polarized TAM (Supplementary Fig. 3A, B).
 − APC cells showed an equal distribution in both neoplastic and non-neoplastic cells.

These findings reflect a neoplastic environment with a reduced immune surveillance (*i.e.* higher PD-L1 expression linked by up-regulation of Texh and Treg cells and switching from M1 to M2-TAM). Otherwise, in non-neoplastic tissue an activation of cell-mediated immune response was present with higher number of infiltrating TH and CTL, as previously observed in pre-neoplastic lesions and in spontaneously regressing HPV-lesions [22].

In HPV-negative tumors no differences between neoplastic and matched non-neoplastic tissues were appreciated regarding the activation of immune evasion mechanisms and the distribution of the T cells’ subsets, with some samples presenting as “hot cancer” and other as “cold cancer”.

By stratifying our samples according to the site (right vs left) no statistically significative differences were appreciated in the distribution of lymphoid population. This might be due to the small number of cases examined. Nonetheless, in left site cancers, a higher number of Texh and Treg cells along M2-TAM were present, probably reflecting an immunosuppressive TME.

## Discussion

CRC is a highly prevalent tumor and does not present a well-defined etiology; therefore, all factors that might lead to its development, including infection and specifically HPV, due to its peculiar way of transmission, need to be investigated. So far, a total of three studies, including a single case–control study, have been reported in Italy to demonstrate the relationship between HPV and CRC. They utilised the PCR technique for the detection of HPV using E6, E7 and L1 region-specific primers and identified HPV positivity with varying frequencies ranging from 15.8 to 33.3% in CRC and 8.8% in normal samples [23]. The variance observed among these studies could be, at least in part, explained by the heterogeneity of the samples, the detection methods, the geographical differences [14–16, 24–26]. Last but not least, the immune response of each individual may account for HPV clearing [27–30].

In our study, we documented the presence of HPV-DNA in 30% of CRC patients by PCR, mainly in left side and exclusively in pMMR tumors. These findings confirm previous data showing an inverse correlation between tumoral mutational burden and viral load [31].

Regarding the detected genotypes, we confirmed previous findings on Italian population; accordingly, HPV16 was the most frequent one, followed by HPV18 [23]. Interestingly, the HPV18 was identified almost exclusively in adenocarcinomatous samples, supporting previous findings that infection of cells with a glandular differentiation may play an important role in the adenocarcinoma development [32].

To demonstrate the transcription activity of the virus in infected cells, we evaluated the expression of p16, Rb and p53 in neoplastic samples [33, 34]. In fact E6 and E7 oncoproteins induce p16 overexpression that, in turn, inactivates and degrades Rb and p53 [35]. We verified that some of the HPV-DNA negative tumors also presented p16 overexpression. Therefore, in CRC, p16 expression cannot be considered a surrogate marker of HPV infection and integration, and other techniques (i.e. PCR and in situ hybridization) are mandatory to depict virus presence. Different mechanisms independent of HPV infection, such as genetic changes in the *Rb* gene, mutations in the *p16* gene itself and induction of senescence by different oncogenic mechanisms could be involved [36].

As far as p53 and Rb expression is concerned, none of HPV-infected CRC showed expression of both proteins, moreover no inactivating mutations of *P53* and *RB* genes were identified by NGS.

The HPV frequency in our cohort, the detection of viral genome by PCR, the relationship between HPV-DNA, p16 overexpression, p53 and Rb negativity, suggest a potential carcinogenic role of HPV in CRC.

It is reasonable that HPV penetrates in the host by one of the known entry sites (namely oral, genital, anal) [1]. Following, the virus might disappear thanks to clearing mechanisms highly active during the early phase of infection or, alternatively, enter the blood circulation by exosome particles and reach the large bowel [1]. For unknown reasons, in some patients, high-risk HPV infection becomes persistent and provides neoplastic transformation [37]. Trying to address this question, we focused on immune microenvironment and gut microbiota, both known to be involved in cancer development.

Interestingly, in HPV-associated cases we documented a dichotomic distribution of T and macrophages’ populations. In other words, the neoplastic samples showed a reduced immune surveillance, whereas in the normal matched controls an activation of cell-mediated immune response was present. In the formers we observed higher PD-L1 expression coupled by up-regulation of Texh and Treg cells, and switching from M1 to M2 TAM. In the latter, a higher number of CTL and TH cells was identified. A robust CTL and TH cells’ response is needed for the effective HPV clearance, their activation depending on right antigen presentation. It is believable that in non-neoplastic tissues we did not detect HPV as it was eliminated from host cells. In fact, after the entry of HPV, the viral DNA is processed by antigen processing machinery to present to the T cells [3, 5, 10, 37]. Once the antigen is presented, activation of the immune signaling cascades occurs, mediating the production of a wide variety of effector molecules including cytokines and chemokines which ultimately act against the intracellular HPV to eliminate it from host cells [5, 10, 37]. In the early phase of the infection, the virus is highly replicating and primes the immune system [3]. This explains why we found an active TH and CTL response in the microenvironment far from the tumor as previously reported [22]. While T cells are infiltrated to the infected area, there is a cross-talking between HPV-infected tissues, T cells and other resident immune cells. These cell–cell interactions allow the tumor survival. In fact, as the HPV integrated in infected cells, it counters mechanisms to undermine the host’s immune recognition, protect the lesion from immune attack and subsequently promote its growth and ability for sustained immune escape [3]. The virus keeps the expression of its oncogenes at a very low level resulting in low abundance of viral antigens [3]. This further contributes in impairment of antigen processing machinery [3]. As a result, a not efficient antigen recognition and presentation to the T cells does occur. Thus, a poor anti-viral immune response against HPV is generated, providing the virus an escape window from the immune killing mechanisms. Accordingly, our neoplastic samples showed lower number of both CTL and TH cells, as well as many anergic and exhausted T-lymphocytes, reflecting a tumor promoting immune environment. A chronic infection can lead to the exhaustion of CTL by PD-1 overexpression as a consequence of both active suppression and passive defects in signaling and metabolism [5]. In addition, we also documented a major recruitment of Tregs in neoplastic samples [5], thus promoting viral immune evasion and disease pathogenesis. Additionally, HPV-positive samples demonstrated a switch towards M2-TAM that again block a potentially active host immunological response.

As proof of principle, outstanding the important immunomodulatory role of microbiota, we decided to analyze and compare the tissue resident oncobiota of both HPV positive and negative samples. The microbiota structure of HPV-related samples was different from HPV negative tumor tissues. In particular, HPV-positive tumors showed a higher abundance and variety of species with the lower abundance of specific genera, among which the most relevant is *Bacteroides* genus. Notably, we have previously documented in human CRC samples high levels of *Bacteroides* genus that negatively correlated with the IL-9 expression [38]. Although its role in cancer is still unclear, current researches suggest that the IL-9 shows anticancer effects by controlling the T cell function and removing the tumor cells in CRC microenvironment [39, 40]. In particular, Wan et al. has demonstrated that the subset of type 2 innate lymphoid cells (ILC2s) is enriched in CRC and ILC2-derived IL-9 activate CD8 + to inhibit tumor growth, while anti-IL-9 reversed this effect in vivo[39].

*Bacteroides* are the most predominant anaerobic, bile-resistant, gram-negative bacteria present in the gut and are recognized as important modulators of immune system development and homeostasis. This genus includes various species, among which the enterotoxigenic *B. fragilis* that is able to stimulates chronic intestinal inflammation, triggering signal transducers and activators of transcription 3 (STAT3) activation, thus contributing to interleukin (IL)-17 production and Tregs’ accumulation, and so promoting inflammation and colon carcinogenesis [41].

In addition, other species of *Bacteroides* might be involved in the antitumor effects of cytotoxic T-lymphocyte associated antigen 4 (CTLA4) blockade, and remarkably specific T cells’ responses against *Bacteroides fragilis* or *Bacteroides thetaiotaomicron* are associated with greater treatment efficacy with anti CTLA4 [42]. Consequently, it will be interesting to identify the *Bacteroides* species differentially abundant in HPV-related and non-related colon cancer, by more specific approaches, such as PCR or shot-gun metagenomics sequencing. Finally, the PICRUST analysis showed interesting differences in metabolic pathways among samples, such as the enrichment of cancer-supportive biosynthetic functions as the glutamine and glutamate synthesis, pyruvate fermentation and arginine byosinthesis in HPV related tumors compared to HPV negative CRC [43]. Glutamine is a favored resource for cancer metabolism and its depletion occurs in various cancer types, especially in poorly vascularized cancers. Cancer cells use glutamine during glutaminolysis for cell growth and proliferation and by sustaining glutamine production, the microbiome can favor the malignancy and cancer progression [44]. Moreover, pyruvate has previously been shown to support cancer migration and development [45] and, finally, increased arginine levels and later greater levels of NO, dopamine, and serotonin synthesis suggested that the microbiome may impact on cancer cells angiogenesis [46].

Finally, regarding the prevalence of HPV-DNA in left-sided colon cancers, it is important to consider the regional differences within the colon, as they may have significant implications for the tumor microenvironment. The regional specialization within the intestinal immune system is well-documented, with the right colon housing cells with more active immune systems, promoting an efficient immune response, while the left colon predominantly harbors immunosuppressive cells [47]. Therefore, the observed higher frequency of HPV-DNA in left tumors may suggest a possible interplay between HPV infection and the immunosuppressive microenvironment in this region, potentially influencing the oncogenesis and progression of colon cancer. Our findings seem to support this data. Even the gut microbiota composition exhibits variations both spatially and radially from the epithelium to the mucosa to the intestinal lumen[48]. Many studies have found a distinct microbiota composition in proximal and distal colorectal cancers [49, 50]. Of particular interest is the concentration of *Fusobacterium*, a bacterial species linked to inflammation and tumorigenesis, which is highest in the proximal colon compared to the left colon, showing a steady increase from the rectum to the cecum[51]. Furthermore, other studies have identified variations in the abundance of *Prevotella* and *Firmicutes* in patients with different colonic locations[52].

In our study, while we did not observe significant differences in the overall microbiota structure between left and right colon cancer samples, we did note slight variations in the abundance of three genera, including one belonging to the Prevotellaceae family. The distinct presence of certain bacterial taxa may play a role in shaping the tumor microenvironment and immune response, potentially influencing the interaction between HPV and colorectal cancer in specific regions of the colon. These regional differences could offer valuable insights into the complex interactions between HPV, tumoral mutational burden, and the immune microenvironment, warranting further investigation to better understand the pathogenetic role of HPV in colon cancer and its potential therapeutic implications.

Despite the valuable insights gained from our study, it is important to acknowledge certain limitations that may impact the interpretation of our findings. First and foremost, the sample size of our study cohort may be considered relatively small, which can influence the statistical power and generalizability of the results. Larger, multi-centric studies are warranted to further validate our observations and establish stronger associations between HPV infection and colorectal cancer.

Furthermore, our investigation was hindered by the lack of complete and relevant information in the medical records, particularly with regard to sexual behavior. Since sexual transmission is one of the known routes for HPV infection, understanding the sexual history of the participants could have provided additional context and shed light on potential risk factors. Unfortunately, the absence of this data limited our ability to comprehensively assess the impact of sexual behavior on HPV infection and colorectal cancer development.

In addition, as an observational study, we did not have a follow-up period to assess long-term outcomes or disease progression. This absence of follow-up data prevents us from establishing causal relationships or making definitive conclusions regarding the temporal aspects of HPV infection and colorectal cancer development. Regarding the characterization of the microbiota, we utilized 16S RNA sequencing, which provides valuable information about the microbial composition but lacks the resolution for species-level characterization. While this approach offers insights into the overall microbial community, a more detailed analysis at the species level could provide a deeper understanding of the specific microbial interactions and their potential associations with HPV-related CRC. Moreover, the limited number of samples analyzed in our microbiota characterization may have influenced the diversity and representation of the microbial community. Expanding the sample size in future investigations would enhance the robustness and reliability of the microbiota analysis.

## Conclusions

There is a cornucopia of tumor extrinsic and intrinsic mechanisms which can provide resistance to immune control and favors tumor evolution. Our research represents a pivotal study linking for the first-time the persistent HPV infection with the host microbiota-immunity axis, and the CRC development in a geographic area known to report a very high incidence of HPV infection. Some limitations were found during the research; among them the sample size, the lack of complete and relevant information in the medical records (namely sexual behavior) and a long follow-up. Even so, we depicted the real-life situation of CRC patients in a referral Hospital. Thus far, a more comprehensive understanding of the factors modulating the local immunity, including less inquired components (such the IL-9 and the immune cells producing it, such as the innate lymphoid cells) and their correlation with microbiota is required to fine decipher the immune landscape of HPV-related CRC. It is only working from this level of comprehension that innovative treatments would be developed.

### Supplementary Information

Below is the link to the electronic supplementary material.

Supplementary file1 (DOCX 1427 KB)

## Data Availability

The 16S rRNA sequence data have been deposited in the NCBI Gene Expression Omnibus (GEO) repository, access number GSE216589 at https://www.ncbi.nlm.nih.gov/geo/query/acc.cgi?acc=GSE216589
