# Supplementary material for: Immune landscape and oncobiota in HPV-Associated Colorectal Cancer: an explorative study
Source: Clin Exp Med. 2023 Aug 23;23(8):5101–12. doi: 10.1007/s10238-023-01165-3 (PMC10725376; doi:10.1007/s10238-023-01165-3)
Supplement: Supplementary file 1 — Supplementary file1 (DOCX 1427 KB) [file 10238_2023_1165_MOESM1_ESM.docx]

**Supplementary Table 1**. Significant differentially abundant taxa between HPV-positive and HPV-negative CC samples. The table report the Log2FoldChange and adjusted p-values assessed using a paired Wilcoxon signed-rank test.

| baseMean | log2FoldChange | padj |  | |
| --- | --- | --- | --- | --- |
| 331,5779 | 6,703488 | 8,85E-05 | Xanthomonadaceae | Family |
| 68,34988 | 3,545039 | 0,00134 | Caulobacteraceae |  |
| 275,7739 | 2,876898 | 0,044855 | Campylobacteraceae |  |
| 3802,902 | 3,437169 | 0,000611 | Bacteroides | Genus |
| 39,88962 | 9,158531 | 0,000611 | Achromobacter |  |
| 309,0686 | 8,067393 | 0,004371 | Stenotrophomonas |  |
| 60,36088 | 5,009822 | 0,005578 | Brevundimonas |  |
| 32,29745 | 7,623889 | 0,036813 | Fretibacterium |  |
| 60,66979 | 4,176698 | 0,044341 | Cavicella |  |
| 64,52887 | 4,049011 | 0,044341 | Mycobacterium |  |

**Supplementary Figure 1. Linear discriminant analysis (LDA) effect size (LEfSe)** negampys more abundant (LDA score >2) in HPV-positive samples are indicated with in green while pathways more abundant in HPV-negative CC samples (LDA score<-2) are indicated in red. (LDA= Linear discriminant analysis).

**
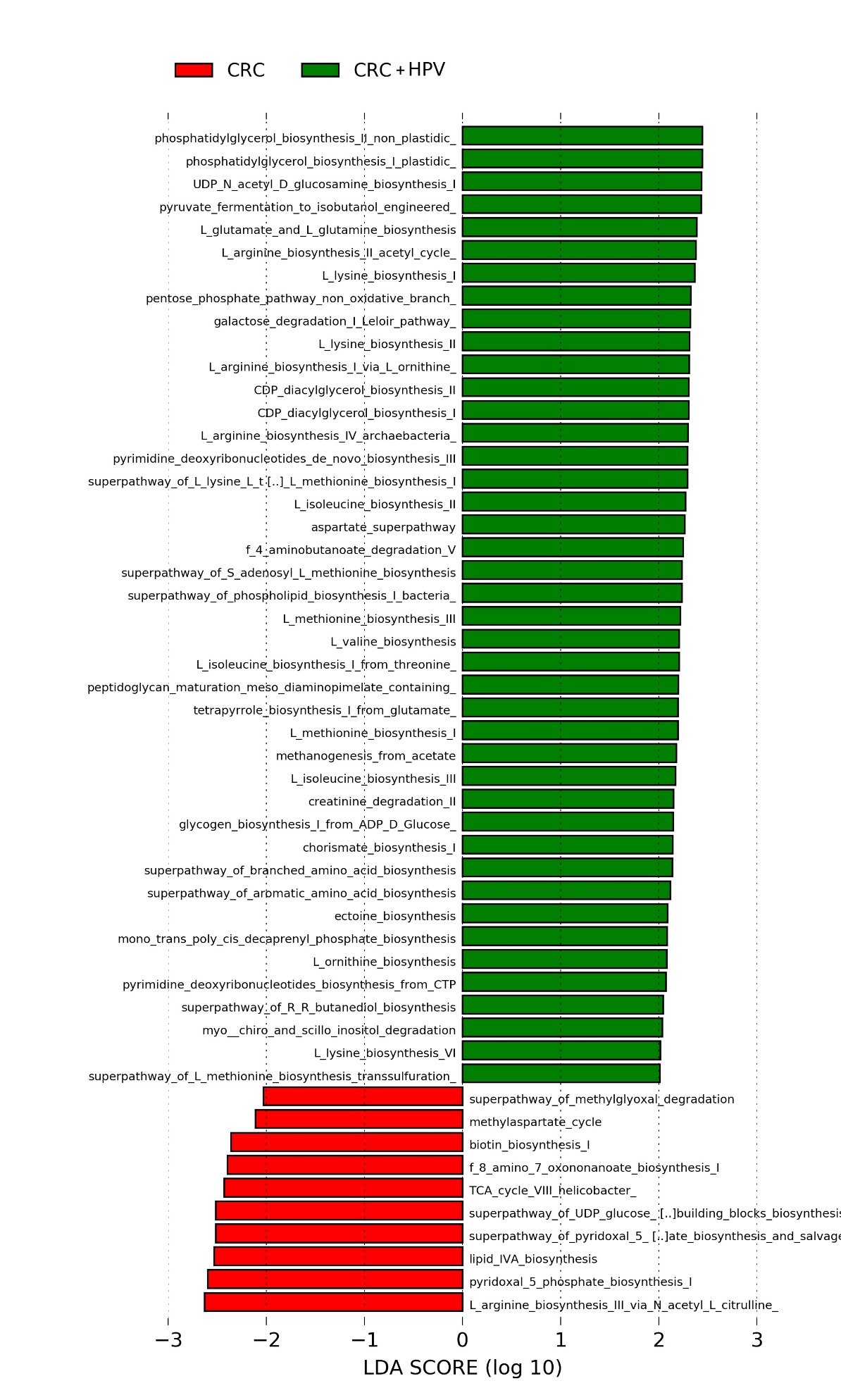
**

**Supplementary Figure 2. Immunological status of HPV-positive neoplastic samples.** Tumor microenvironment shows high number of CD8/PD-1 positive T-exh (A, arrow) and of CD4/CD25 positive Treg lymphocytes (B, circle) and M2 TAM (C). High expression of PD-L1 observed in neoplastic cells (D). A, CD8/PD-1 double stain; B, CD4/CD25 double stain; C, CD163/CD68 double stain; D, PD-L1 stain. Original magnification (O.M.): A, B, C O.M. 20x; D, O.M. 10x.


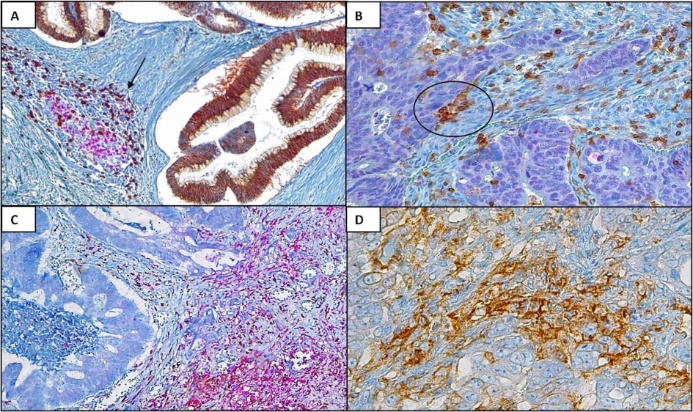


**Supplementary Figure 3**. **Immunological status in matched non neoplastic samples.** Matched non neoplastic samples show high levels of CD8-positive (A) and CD4-positive lymphocytes (A, inset) and low number of CD8/PD-1 positive T-exh (B, circle) and CD4/CD25 positive Treg lymphocytes (B, inset, arrow). M1 polarization of TAM is observed (C). A, CD4 stain; A inset, CD8 stain; B, CD8/PD-1 double stain; B inset, CD4/CD25 double stain; C, CD68/CD163 double stain. Original magnification (O.M.): A, A inset, B, B inset, O.M 20x, C, O.M. 40x.

**
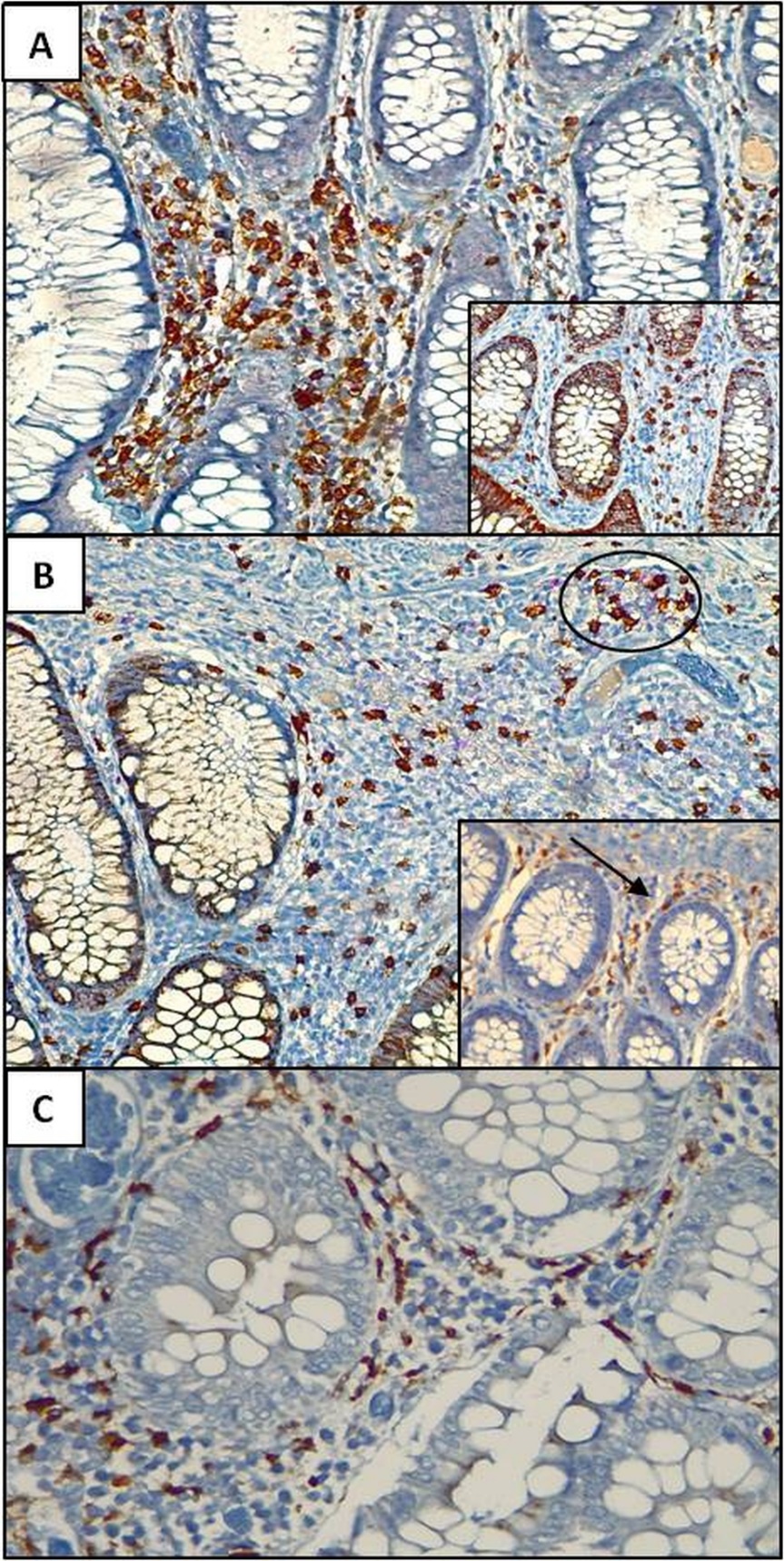
**
